# Malformations vasculaires au cours du syndrome de Williams-Beuren: à propos de trois nouvelles observations

**DOI:** 10.11604/pamj.2016.23.38.7135

**Published:** 2016-02-10

**Authors:** Hicham Sator, Fatima Ezzahra Rhouni, Ibitihale Benjouad, Fatima Ezzahra Rhouni, Ibitihale Benjouad, Rachida Dafiri, Latifa Chat

**Affiliations:** 1 Service de Radiologie, Hôpital des Enfants, CHU Ibn Sina, Rabat, Maroc

**Keywords:** Williams Beuren, sténose supra aortique, angioscanner, angio IRM, Williams Beuren, supra aortic stenosis, CT angiography, MR angiography

## Abstract

Le syndrome de Williams-Beuren est une maladie génétique rare, il associe classiquement une dysmorphie faciale assez spécifique, des malformations cardiovasculaires et un profil neuropsychologique particulier. Nous rapportons les observations de trois enfants atteints du syndrome de Williams-Beuren en insistant surtout sur les anomalies vasculaires observées sur l'angio-scanner et angio-IRM.

## Abstract (en)

The Williams-Beuren syndrome is a rare genetic disease. It combines classically specific facial dysmorphism, cardiovascular malformations and specific neuropsychological profile. We report three cases of Williams-Beuren syndrome in children with particular emphasis on vascular abnormalities observed on CT angiography and MR angiography.

## Introduction

Le syndrome de Williams-Beuren est une maladie génétique rare due à une microdélétion au niveau du bras long du chromosome 7. Il est également réparti entre les deux sexes et associe classiquement une dysmorphie faciale assez spécifique, des malformations cardiovasculaires et un profil neuropsychologique particulier. Nous rapportons les observations de trois enfants atteints de syndrome de Williams-Beuren âgés entre 4 et 15 ans en insistant surtout sur les anomalies vasculaires observées sur l'angio-scanner et l'angio-IRM.

## Patient et observation

### Observation n^°^1

**Figure 1:**
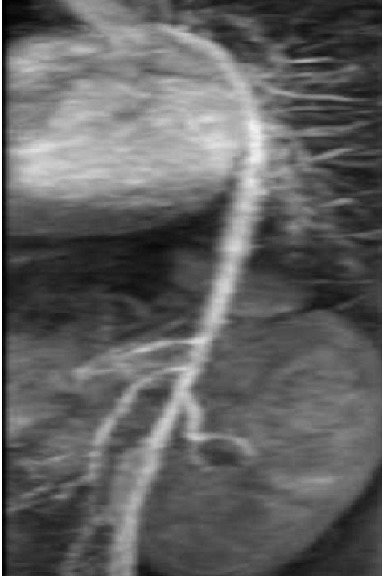
Angio IRM 3D: aorte thoraco-abdominale grêle

**Figure 2:**
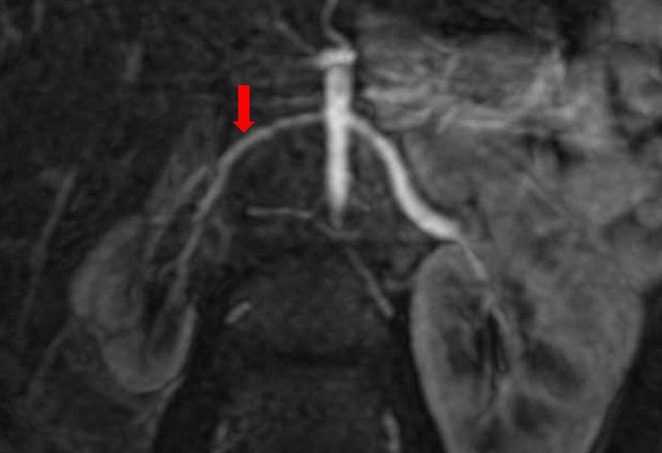
Angio IRM 3D: artère rénale gauche de calibre réduit (flèche) avec rein gauche de petite taille, noter l'aspect normale de l'artère rénale et du rein droits

Youssef, âgé de 4 ans, a été adressé à notre service pour infection urinaire, il est né par voie basse à 40 semaines d'aménorrhée, la marche a été acquise à 18 mois. Il avait souffert de plusieurs épisodes de diarrhées avec perte de poids. A l'examen clinique il présentait une dysmorphie faciale qui comportait une racine du nez aplatie, une grande bouche avec une lèvre inférieure éversée et un ‘dème périorbitaire. La tension artérielle était à 170/90 mmHg. Il était apyrétique et l'examen neurologique retrouvait une hypotonie prédominant aux membres inférieurs. Devant la dysmorphie faciale, le syndrome de Williams-Beuren a été suspecté et une atteinte des artères rénales dans le cadre de ce syndrome à l'origine de l'hypertension artérielle a été évoquée. L’écho-Doppler rénal a été non concluant. Cependant l'angio-IRM a montré une aorte thoraco-abdominale perméable de calibre réduit dans son ensemble (Figure 1), l'artère rénale droite était grêle mais perméable (Figure 2), le rein droit était de petite taille avec une bonne différenciation et une néphrographie normale. Le rein et l'artère rénale gauches étaient normaux. En outre l'artère pulmonaire droite était grêle. Les artères viscérales avaient un calibre normal. L’étude du caryotype par fluorescence in situ hybridization (FISH) a retrouvé la microdélétion du chromosome 7 spécifique du syndrome de Williams-Beuren.

### Observation n^°^2

**Figure 3:**
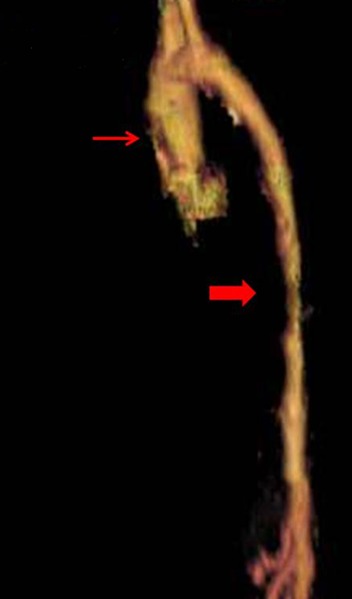
Angioscanner thoraco-abdominale avec reconstruction 3D: sténose supra valvulaire de l'aorte ascendante (flèche), rétrécissement de l'aorte abdominale (flèche large)

Soufiane, âgé de 15 ans, a été suivi depuis l’âge de 3 ans pour le syndrome de Williams-Beuren qui a été diagnostiqué à l'occasion d'un retard des acquisitions psychomotrices. Dans le cadre de son suivi une échocardiographie a suspectée une sténose supra valvulaire de l'aorte, elle a été complétée par un angio-scanner thoraco-abdominal. Cet angio-scanner a montré une sténose aortique supra valvulaire modérée qui a été associée également à un rétrécissement régulier étagé de l'aorte descendante et abdominale (Figure 3). Les troncs supra aortiques étaient normaux. Il n'a pas été noté d'anomalie des artères pulmonaires ou d'anomalie du retour veineux pulmonaire.

### Observation n^°^3

**Figure 4:**
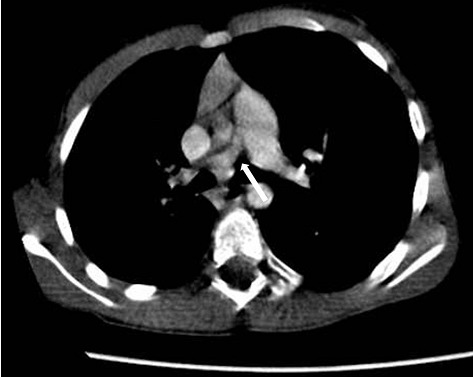
Angioscanner thoracique: sténose de la racine de l'artère pulmonaire droite (flèche)

Adam, âgé de 5 ans, a été connu porteur du syndrome de Williams-Beuren depuis l’âge de 18 mois suite à la constatation des parents d'une dysmorphie faciale et d'une irritabilité. L'angio-scanner thoracique et des troncs supra aortiques a montré une sténose au niveau de la racine de l'artère pulmonaire droite associée une aorte ascendante et abdominale grêle (Figure 4). Il a été objectivé également une sténose peu serrée au niveau de l'origine de la carotide primitive droite. Par ailleurs, il n'a pas été objectivé d'anomalie au niveau de l'artère carotide gauche ou au niveau des artères sous clavières.

## Discussion

Le syndrome de Williams-Beuren est une maladie génétique rare due à une micro délétion au niveau du bras long du chromosome 7 dans la région 7q11.23 comportant entre 17 et 25 gènes dont celui codant pour l’élastine, un constituant essentiel du tissu artériel extracellulaire. Le syndrome de Williams-Beuren est également réparti entre les deux sexes et associe une dysmorphie faciale et diverses malformations cardiovasculaires avec un profil neuropsychologique particulier. Le diagnostic est posé en général vers l’âge de quatre ans le plus souvent suite à une cardiopathie. La dysmorphie faciale est très caractéristique comportant un front large, un menton pointu, une racine du nez aplatie avec une extrémité bulbeuse, une grande bouche avec lèvre inférieure large et tombante et un strabisme. Cette dysmorphie est qualifiée de «faciès d'elfe». Les anomalies cardiovasculaires sont présentes chez environ 80% des patients porteurs du syndrome de Williams-Beuren, caractérisées par des rétrécissements artériels survenant sur de nombreux vaisseaux essentiellement l'aorte, les artères pulmonaires et les artères rénales [1]. Des atteintes des artères coronaires et des artères carotides responsables d'infarctus myocardiaque ou cérébral ont été rapportées [2, 3]. Les anomalies vasculaires les plus fréquentes sont la sténose aortique supra valvulaire et les sténoses de l'artère pulmonaire et de ses branches. Ces deux anomalies coexistent chez environ le tiers des patients [4]. L'atteinte aortique peut être plus diffuse avec des sténoses plus ou moins continues le long de l'aorte. Plus rarement une atteinte valvulaire aortique ou mitrale, des défauts septaux ou une tétralogie de Fallot peuvent être observées. L’échographie cardiaque révélant une sténose aortique supra valvulaire ou une sténose pulmonaire peut être réalisée en période néonatale ou dans la petite enfance devant la découverte d’‘un souffle cardiaque. Elle doit être réalisée systématiquement lors du diagnostic du syndrome de Williams-Beuren.

L'atteinte rénale peut expliquer l'hypertension artérielle, toutefois, 50% des patients hypertendus ne présentent pas d'anomalie rénale. Dans notre première observation le caractère grêle de l'artère rénale droite, contrastant avec une néphrographie et une artère rénale gauche normales, ne permettait pas à lui seule d'expliquer l'hypertension artérielle observée chez notre patient et probablement d'autres mécanismes entrent en jeu. L’évaluation échographique non invasive de la compliance de la carotide chez les patients hypertendus atteints du syndrome Williams-Beuren n'a pas révélé d'altération de ses propriétés élastiques, malgré un épaississement pariétal avéré. L'hypertension artérielle ne semble donc pas pouvoir être attribuée à une altération de la compliance artérielle des vaisseaux. Sur le plan histologique, les artères des patients atteints du syndrome Williams-Beuren présentent un épaississement du média résultant à la fois d'une augmentation du nombre et des couches des cellules musculaires lisses et d'une néo synthèse de collagène associé à des lésions de l'intima, le tout évoluant vers une occlusion de la lumière artérielle. Il est admis que la sténose aortique supra valvulaire s'aggrave avec le temps en particulier dans les cinq premières années de la vie, nécessitant une surveillance régulière [5]. Pourtant une régression spontanée a été rapportée chez 16% des cas [4]. Par contre la plupart des sténoses artérielle pulmonaire ou de ses branches ont un bon pronostic et régressent spontanément et ne nécessitant une intervention chirurgicale que dans des cas exceptionnels. Le profil neurocognitif comprend un QI moyen de l'ordre de 60 avec des extrêmes allant de 40 à 100. Le langage, souvent considéré comme préservé, reste très superficiel utilisant des phrases stéréotypées imitant l'intonation des adultes. Des anomalies de l'appareil urinaire ont été rapportées chez 20% à 35% des patients. Les diverticules de la vessie sont les plus fréquents et peuvent être dus à la carence en élastine. Une néphrocalcinose peut compliquer l'hypercalcémie initiale [6]. Malgré la présence d'une infection urinaire chez notre premier patient, aucun de nos patients ne présentait d'anomalie des voies urinaires. A la naissance les enfants atteints de syndrome Williams-Beuren présentent des troubles digestifs pouvant entraîner un retard de croissance. Une hypercalcémie et une hernie inguinale ou ombilicale sont notées de façon inconstante. La prise en charge des enfants atteints de ce syndrome doit se faire dans un cadre multidisciplinaire. Le facteur pronostique prédominant est vraisemblablement l'atteinte cardiovasculaire qui doit être suivie par une équipe spécialisée. La stratégie thérapeutique est surtout de nature chirurgicale. Le type d'intervention choisi dépend de l'importance de la sténose et doit tenir compte du fait que l'ensemble de la paroi artérielle est affectée.

## Conclusion

Le syndrome de Williams-Beuren est une maladie génétique rare, caractérisée par la présence de plusieurs malformations vasculaires dont la gravité détermine le pronostic. Le rôle de l'imagerie est important pour l’évaluation de ces malformations.
